# Does the Position of the Mandibular Third Molar Have an Effect on the Lingual Bone Morphology? A Cone Beam Computed Tomography Evaluation

**DOI:** 10.3390/diagnostics15182401

**Published:** 2025-09-20

**Authors:** Ezgi Yüceer-Çetiner, Özgür Sakarya, Attila Vural, Dilara Kazan

**Affiliations:** 1 Department of Oral and Maxillofacial Surgery, School of Dental Medicine, Bahçeşehir University, Istanbul 34357, Türkiye; dilara.kazan@bau.edu.tr; 2 Department of Oral and Maxillofacial Surgery, Faculty of Dentistry, Biruni University, Istanbul 34015, Türkiye; dtozgursakarya@gmail.com (Ö.S.); vural.attila@gmail.com (A.V.)

**Keywords:** cone beam computed tomography, impacted teeth, lingual morphology, mandibular third molar

## Abstract

**Background/Objectives:** This study aimed to investigate the association between the position of impacted mandibular third molars and the morphology of the lingual cortical bone using cone beam computed tomography (CBCT), and to determine how impaction depth and angulation influence the risk of lingual cortical perforation. **Methods**: CBCT scans of 120 impacted mandibular third molars from 71 adult patients were retrospectively evaluated. Teeth were classified based on Pell & Gregory’s and Winter’s classifications. Lingual cortical morphology was categorized as undercut, parallel, slanted, or round. The relationship between the root apex and the lingual plate was classified as non-contact, contact, or perforating. Linear measurements included cortical lingual bone thickness and the distance from the apex to the outer surface of the lingual cortex. **Results**: Lingual bone morphology showed significant associations with both impaction depth and angulation, with parallel morphology more common in deeper and more angulated impactions. Lingual cortical perforation was observed in approximately 30% of the teeth, predominantly at the apex, with horizontal and deeply impacted molars (Class II, Level C) representing the highest-risk configurations. Although cortical thickness and apex-to-cortex distance were significantly smaller in apically perforated cases, no definitive threshold could be established, and these parameters were insufficient as standalone predictors at the cementoenamel junction or mid-root levels. **Conclusions**: Tooth angulation and impaction depth are significant predictors of lingual bone morphology and perforation risk. CBCT imaging is therefore recommended beyond low-risk cases (Level A, Class I, vertical) to improve preoperative planning, strengthen informed consent, and guide surgical strategies aimed at minimizing complications and enhancing patient safety.

## 1. Introduction

The surgical extraction of third molars, being the most commonly impacted teeth in the jawbones, is associated with various complications such as pain, trismus, neurosensory disturbances in the inferior alveolar nerve and lingual nerve, bleeding, fracture of lingual plate, and displacement of root or bone fragments into the lingual soft tissue [1,2]. Although nerve injuries, lingual plate fractures, or root fragment displacements are not common complications, they can lead to severe conditions such as hemorrhage or infection of the anatomical spaces when they do occur [3,4,5,6]. Such serious complications can arise from inadequate assessment of factors such as lingual bone thickness, proximity of the tooth root to the lingual bone and soft tissues, perforation of the lingual bone by the tooth root, and the relationship between the tooth root and the mandibular canal during the preoperative evaluation of mandibular impacted third molars [7,8].

Determining the risk factors associated with complications during and after impacted third molar surgery primarily relies on preoperative radiographic evaluations. While panoramic radiography is widely used to observe anatomical structures and detect pathological lesions in both the maxilla and mandible, it is also routinely employed to assess the depth and angulation of mandibular impacted third molars, discern their topographic relationship with the inferior alveolar canal, and conduct preoperative risk assessment for impacted third molar surgery [9,10,11]. However, this two-dimensional technique has drawbacks such as unequal magnifications, artifacts, potential distortions, and superimposition of images, which impede the detailed and accurate evaluation of the mandible’s local anatomy and lingual bone integrity [7,12]. Cone beam computed tomography (CBCT), which allows thorough examination of teeth, jaw, and their adjacent tissues, is also frequently used in the examination of mandibular third molar teeth and is capable of tolerating data loss and addressing the inadequacies of panoramic radiography in buccolingual assessment [9,10,11,12,13,14]. The literature contains studies where factors such as the morphology or thickness of the lingual cortex, the position of impacted mandibular third molars, and the relationship between the roots and lingual bone can lead to lingual bone fractures, displacement of roots into anatomical spaces, and potential lingual nerve damage during or after the impacted third molar extractions. However, there is a lack of studies comprehensively analyzing the topographic relationship between the roots of impacted mandibular third molars and lingual bone in the literature [15,16,17,18,19].

The purpose of this study was to assess the topographic relationship between the root apex of impacted mandibular third molars and the lingual cortical bone using CBCT images, and to investigate the influence of tooth position, impaction depth, and angulation on lingual bone morphology and the risk of cortical perforation.

## 2. Materials and Methods

This study followed the Declaration of Helsinki on medical protocol and ethics and was approved by the Ethical Review Board of Biruni University Non-invasive Clinical Research Ethics Committee (no: 2023/78-11; date: 21 February 2023).

### 2.1. Study Design and Sample

The study included 120 impacted mandibular third molars from 71 patients (27 female, 34 male) who had completed skeletal development. Written informed consent was obtained from all participants. An a priori power analysis was conducted using G*Power v3.1.9.6 (Heinrich-Heine-Universität Düsseldorf, Düsseldorf, Germany), which showed that a total sample size of 111 would be required to achieve adequate power (α = 0.05; 1 − β = 0.95). Our study sample (*n* = 120) exceeded this threshold, confirming that the analysis was sufficiently powered.

The inclusion criteria were as follows:

- Age ≥ 18 years.
- Presence of mandibular third molars that were impacted.
- CBCT images including the entire mandibular body.

The exclusion criteria were as follows:

- Patients under 18 years of age.
- Cases with the presence of caries, bone destruction, or any other pathologies.
- Missing first or second molars in the same quadrant.
- Images with artifacts or insufficient diagnostic quality.

### 2.2. CBCT Image Acquisition and Analysis

All CBCT images were taken by the same technician using the same radiographic equipment (Sirona Galileos Comfort Plus CBCT device, Bemshein, Germany) at 98 kV, 6 mA, and a voxel size of 0.25 mm. The field of view (FOV) of the images assessed in this study was 15 × 15 cm. The Sidexis 4 software (version 4.3.1, Bemshein, Germany) was used to convert the original Digital Imaging and Communication in Medicine (DICOM) format.

### 2.3. Radiological Assessments and Parameters

**Figure 1:**
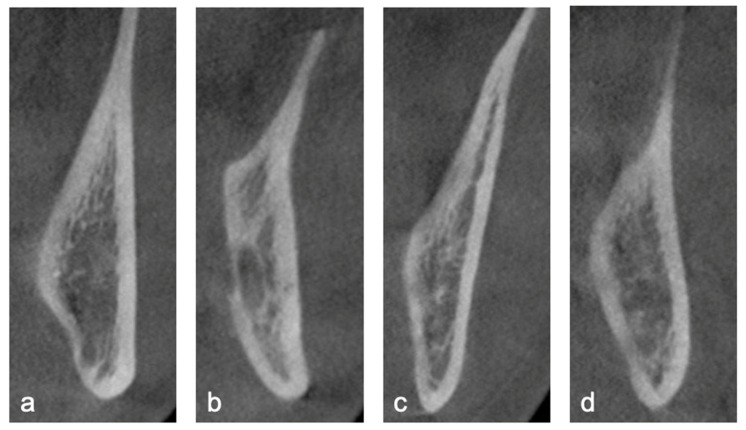
Lingual plate morphology at the level of root apex identified on sagittal CBCT section. (**a**) Type U, undercut on the lingual side, (**b**) Type P, parallel to the buccal plate, (**c**) Type S, slanted with buccolingual width reduced on the lingual side, (**d**) Type R, round shape on the lingual side.

**Figure 2:**
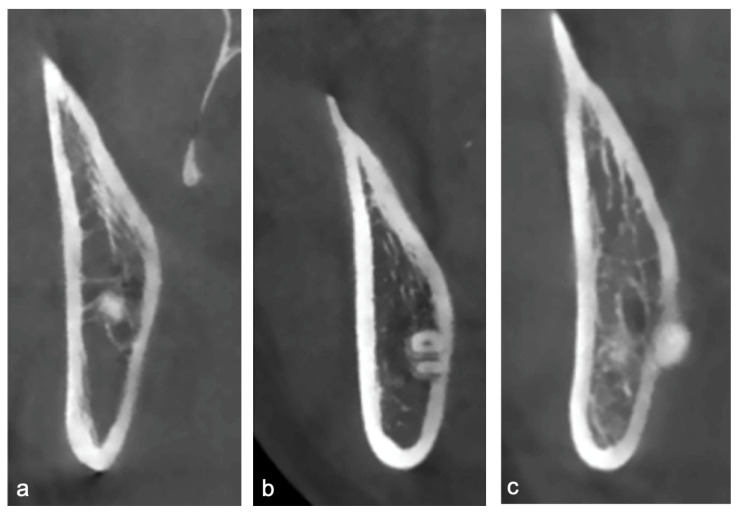
Topographic relationship between the root apex and the lingual plate. (**a**) non-contact, (**b**) contact, (**c**) perforating.

The following parameters were evaluated:

- Impaction Classification: Each impacted third molar was classified based on Pell & Gregory’s classification (for ramus relationship and occlusal level) and Winter’s classification (for angulation).
- Lingual Plate Morphology: Morphology of the lingual cortical bone at the level of the root apex was classified as undercut, parallel, slanted, or round (Figure 1).

- Root Apex Position: The topographic relationship between the root apex and the lingual plate was categorized as non-contact, contact, or perforating (Figure 2). The presence or absence of lingual cortical perforation was assessed at three anatomical levels of each impacted mandibular third molar: the cementoenamel junction (CEJ), mid-root, and root apex.
- Linear Measurements: Cortical lingual bone thickness (at the apex level) and distance from the root apex to the outer surface of the lingual plate were measured as morphometric parameters.

**Figure 3:**
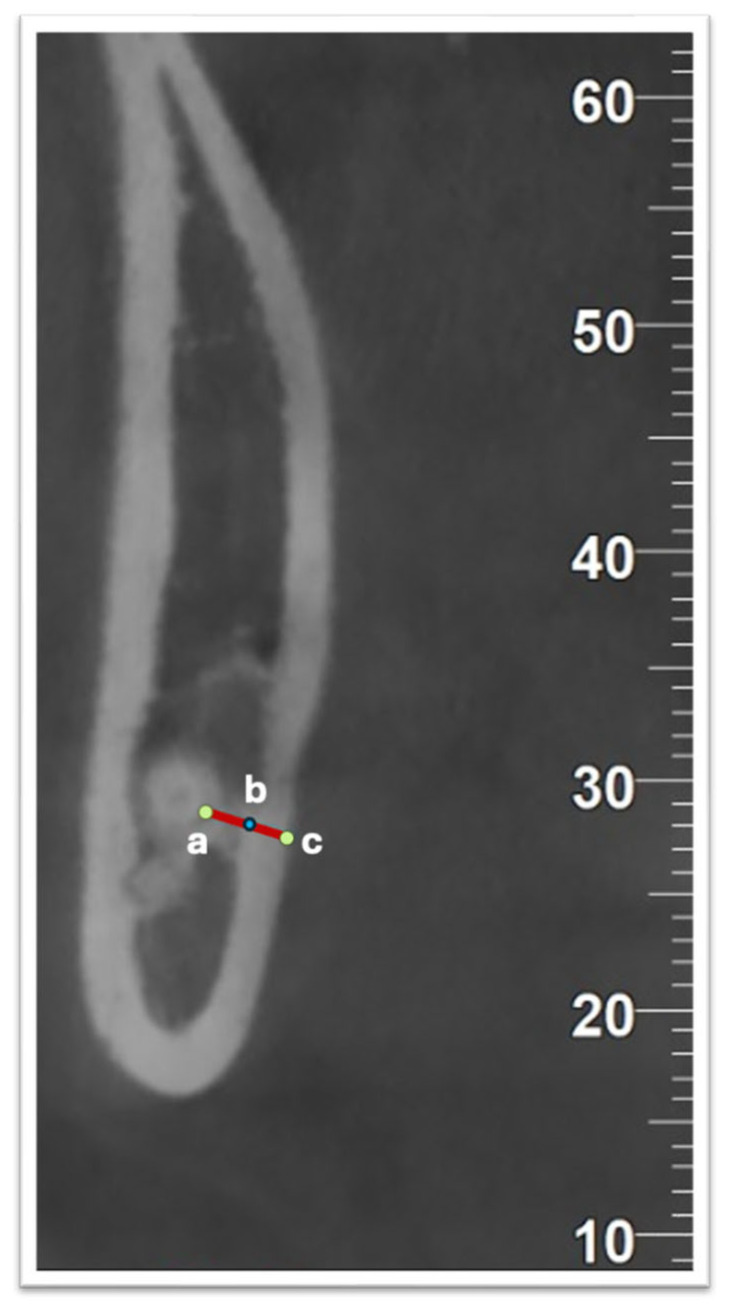
CBCT cross-sectional image demonstrating measurement methodology. Point a represents the apex of the root located most lingually, point b the inner surface of the lingual cortical plate, and point c the outer surface of the lingual cortical plate. The b–c line indicates the thickness of the cortical lingual bone, while a–c represents the distance from the root apex to the outer surface of the lingual plate.

To determine the relevant measurements, the most lingually positioned root apex of each impacted third molar was identified using axial CBCT images. In multi-rooted teeth, the root located most lingually was selected. The image slice showing the most distal extent of the root apex was used for further analysis. The images were then examined sequentially in mesial and distal directions (in 0.1 mm intervals) to locate the apex precisely. Once the clearest view of the apex was selected, measurements were made on the cross-sectional plane. A horizontal reference line was drawn from the most lingual point of the root apex, intersecting the inner and outer surfaces of the lingual cortical bone. Using the software’s measurement tools, the thickness of the lingual plate and the distance from the root apex to its outer surface were calculated. Each measurement was repeated three times, and the mean value was recorded (Figure 3).

### 2.4. Statistical Analysis

Statistical analyses were performed using IBM SPSS Statistics 22 software (IBM Corp., Armonk, NY, USA). The normality of distribution for each parameter was assessed using both the Kolmogorov–Smirnov and Shapiro–Wilk tests, and it was determined that the data did not follow a normal distribution. Both tests were employed as they provide complementary and reliable validation of normality assumptions, especially for sample sizes between 50 and 200. Since the normality assumption was violated, non-parametric tests (Kruskal–Wallis and Mann–Whitney U) were used instead of parametric alternatives such as ANOVA or *t*-tests. Descriptive statistical methods (mean, standard deviation, frequency) were used to summarize the data. For comparisons of quantitative variables across more than two groups, the Kruskal–Wallis test was applied, and post hoc analyses were conducted using Dunn’s test to identify the source of significant differences. Pairwise comparisons between two independent groups were performed using the Mann–Whitney U test. For the analysis of categorical variables, the Chi-square test, Fisher-Freeman-Halton exact test, and Yates’ continuity correction were employed where appropriate. A *p*-value of less than 0.05 was considered statistically significant.

## 3. Results

Based on the inclusion and exclusion criteria, this study was conducted with 120 wisdom teeth of a total of 71 cases, 27 (52.1%) females and 34 (47.9%) males, aged between 19 and 69 years. The mean age was 31.5 ± 10.7 years. According to Pell–Gregory’s classification based on the distance to the ramus, 37.5% of the third molars were Class I, 43.3% were Class II, and 19.2% were Class III, with respect to impaction depth relative to the occlusal plane, 38.3% were Level A, 31.7% Level B, and 30% Level C. Regarding angulation based on Winter’s classification, 27.5% were mesioangular, 55.8% were vertical, 15.8% were horizontal, and 0.8% were buccolingual. In terms of lingual bone morphology, 29.2% of the samples exhibited an undercut configuration, 28.3% were parallel, 30% were slanted, and 12.5% were round. As for the position of the root apex relative to the lingual plate, 35.8% were in contact, 34.2% were non-contact, and 30% were perforating. Perforation at different root levels was observed in 9.2% at the cementoenamel junction (CEJ), 25% at the mid-root, and 29.2% at the apex (Table 1). No statistically significant differences were found in any of the evaluated parameters between the sexes (*p* > 0.05).

A significant difference among the morphologies of the lingual bone was observed according to Pell & Gregory’s classification, with parallel morphology in Class I (11.1%) being significantly less frequent than in Class II (34.6%) and Class III (47.8%) (*p* = 0.020), as well as in Level A (8.7%) being significantly less frequent than in Level B (34.2%) and Level C (47.2%) (*p* = 0.008). There was no statistically significant difference between the apex positions and Pell & Gregory’s classifications (both available space and impaction depth) (*p* > 0.05). Perforation rates at the CEJ (*p* = 0.058) and mid-root (*p* = 0.221) showed no significant differences according to ramus distance. However, the incidence of apical perforation was significantly less frequent in Class I (15.6%) than in Class II (42.3%) (*p* = 0.014). Additionally, apex positions showed no significant difference based on impaction depth (*p* = 0.110). However, perforation at the CEJ was significantly more common in Level C (27.8%) than in Level A (0%) and Level B (2.6%) (*p* = 0.001). There were no significant differences in mid-root (*p* = 0.968) or apex (*p* = 0.572) perforation rates across impaction levels (Table 2 and Table 3).

Lingual bone morphology also varied significantly by tooth angulation according to Winter’s classification (*p* = 0.001). The rate of parallel morphology was significantly lower in vertical impactions (10.4%) compared to mesioangular (45.5%) and horizontal (57.9%) positions. There was no statistically significant difference between the apex locations and the position of the teeth according to Winter classification (*p* > 0.05). However, CEJ-level perforation was significantly more common in horizontally impacted molars (36.8%) compared to mesioangular (6.1%) and vertical (1.5%) (*p* = 0.001). Mid-root and apex perforation rates showed no significant differences by angulation (*p* > 0.05) (Table 4).

The mean lingual cortical bone thickness was 0.86 ± 0.80 mm, and the mean distance between the root apex and the outer surface of the lingual plate was 1.27 ± 1.73 mm. No statistically significant differences in cortical thickness or apex-to-lingual plate distance were found between teeth with and without perforation at the CEJ or mid-root levels (*p* > 0.05). However, both parameters were significantly smaller in teeth with apical perforation compared to those without (*p* = 0.001 for both comparisons). There were no significant associations between perforation site and lingual bone morphology (*p* > 0.05).

## 4. Discussion

During mandibular third molar removal, perforation of the lingual cortical bone poses significant clinical risks, including hemorrhage, lingual plate fracture, infection of anatomical spaces, and potential nerve injury. In our study, such perforation was observed in approximately 30% of impacted third molars, a finding consistent with Wang et al. (2016), who reported a 32.97% perforation rate using CBCT [8]. However, the literature shows considerable variation. Emes et al. (2014) identified lingual perforation in only 4 of 32 cases, while Tolstunov et al. (2016) reported a much higher rate of 65.5% [20,21]. These differences mainly reflect variation in sample size, case mix (distribution of impaction types and depths), imaging modality, and the definitions of ‘perforation’. Reporting rates by impaction type and imaging method—and using consistent CBCT criteria—would improve comparability across studies and better inform preoperative counseling and surgical planning. Although lingual cortical thickness was significantly reduced in perforated cases, no definitive threshold value in millimeters could be established. This contrasts with studies that attempted to define cutoff values, but such inconsistency likely stems from heterogeneity in voxel size, measurement planes, and observer calibration across CBCT protocols. Our results, therefore, reinforce that cortical thickness, while informative, should be interpreted alongside tooth angulation and impaction depth. This supports the concept of a multifactorial anatomical risk profile, rather than thickness being a reliable standalone predictor.

Our results demonstrated that lingual bone morphology is significantly influenced by both the impaction depth and angulation of mandibular third molars. According to the Pell & Gregory classification based on the available space anterior to the mandibular ramus, the presence of parallel lingual bone morphology was significantly less frequent in Class I impactions (11.1%) compared to Class II (34.6%) and Class III (47.8%). This suggests that as the available retromolar space decreases, the lingual cortex is more likely to adapt a parallel configuration, potentially due to the spatial constraints and long-standing anatomical adaptation in deeper impactions. Similarly, when evaluating vertical impaction depth based on the occlusal plane, parallel morphology was observed significantly less frequently in Level A impactions (8.7%) compared to Level B (26.3%) and Level C (19.4%). These findings reinforce the notion that more deeply embedded third molars are associated with altered lingual bone morphology, which may contribute to surgical difficulty and elevated risk of complications such as root displacement or lingual plate fracture [7,8]. In terms of tooth angulation, a statistically significant relationship was also found, with vertical impactions showing the lowest rate of parallel lingual bone morphology (10.4%), compared to mesioangular (45.5%) and horizontal impactions (58.9%). These results indicate that angulated molars may exert different mechanical pressures on the surrounding bone, which may contribute to the development of the formation of a parallel lingual cortex. Tooth angulation should be considered in preoperative risk evaluation. Our findings are in line with those of Huang et al. (2020), who similarly reported that horizontally impacted third molars are at greater risk for lingual cortical disruption due to their unfavorable angulation and deeper impaction levels [7]. On the other hand, vertical impactions were significantly less likely to be associated with parallel morphology or cortical perforation, suggesting a relatively safer profile for surgical removal. Preoperative evaluation protocols should therefore include CBCT imaging, especially in cases of mesioangular or horizontal impactions, to better visualize bone morphology, assess the risk of lingual plate perforation, and minimize the chance of intraoperative complications.

Our findings also emphasize the association between third molar position and the risk of lingual cortical perforation at different root levels—namely, the cementoenamel junction, mid-root, and apex. In the Pell & Gregory classification, a significant difference was observed only at the apex, where Class I impactions had a lower perforation rate (15.6%) compared to Class II (42.3%), suggesting that deeper impactions increase the likelihood of apical perforation [6,15]. Similarly, CEJ-level perforation was significantly more frequent in Level C impactions (27.8%) than in Level A (0%) and Level B (2.6%), indicating that vertical depth of impaction may contribute to coronal-level cortical vulnerability. According to Winter’s classification, horizontal impactions showed the highest rate of CEJ perforation (36.8%), significantly exceeding that of mesioangular (6.1%) and vertical positions (1.5%). Halder et al. (2023) reported that lingual cortical thickness varied with angulation, being thinner in certain groups, such as mesioangular impactions [22]. Similarly, a recent study comparing panoramic radiography with CBCT identified angulation and impaction type as risk factors [23]. This finding suggests that tooth angulation, particularly in horizontally positioned third molars, may be associated with decreased lingual cortical bone thickness at the CEJ level, thereby increasing the risk of cortical plate fracture during surgical extraction [7]. Notably, despite the observed associations between tooth position and lingual morphology, no statistically significant differences were found between lingual morphology and perforation sites. This also indicates that while tooth position affects bone shape, it may not directly predict the site of cortical perforation will occur, and further multifactorial analysis may be needed to explore other determinants such as cortical thickness, root curvature, or bone density. These findings are consistent with and further support the earlier observation that deeper and more angulated impactions (particularly horizontal and mesioangular) are associated with more challenging anatomical relationships and greater surgical risk.

This study also examined whether lingual cortical bone thickness and the apex-to-outer surface of the lingual plate, as morphometric parameters, were associated with perforation at different root levels. No significant differences were observed between teeth with and without perforation at the CEJ, mid-root, or apex levels, indicating that these measurements alone may not reliably predict perforation risk. Instead, factors such as tooth angulation and impaction depth appear to play a more decisive role, which is consistent with our previous findings. In our study, the mean distance between the apex and the outer surface of the lingual plate was 1.27 mm, aligning closely with Emes et al. (2015)’s findings (1.03 mm), and further supporting the notion that impacted third molar roots lie in close proximity to the lingual plate [20]. The average lingual cortical bone thickness in our sample was 0.86 mm, which is higher than the value reported by Momin et al. (2013) (0.68 mm) but smaller than that of Ge et al. (2016) (1.54 mm) [18,24]. These variations may stem from differences in imaging modalities, measurement levels, or sample selection. Notwithstanding these methodological nuances, both studies converge on the need to contextualize cortical measurements with impaction depth and angulation and support the superiority of CBCT over panoramic radiography for accurate risk stratification before third molar surgery.

Importantly, the patterns of lingual cortical perforation identified in this study provide critical guidance for preoperative planning and the informed consent process. The significantly higher rates of perforation in horizontally and deeply impacted molars (Class II, Level C), together with the overall apical perforation rate of nearly 30%, indicate that complications such as lingual plate fracture, hemorrhage, displacement of root fragments, or lingual nerve injury are not uncommon. These risks should therefore be explicitly communicated to patients during consent discussions. While panoramic radiography may be sufficient in low-risk cases such as Level A, Class I, and vertically impacted molars, CBCT evaluation should be considered essential in all other scenarios to ensure accurate risk stratification and safe surgical planning. Although lingual cortical thickness or apex to lingual plate were not consistently associated with perforation risk at all root levels, their evaluation on CBCT should not be overlooked. In particular, reduced thickness at the apical level may indicate vulnerability; however, no definitive threshold value in millimeters can be proposed based on the current findings. Therefore, cortical thickness should be interpreted in conjunction with other anatomical predictors such as tooth angulation and impaction depth when assessing surgical risk. Incorporating these anatomical predictors into routine preoperative assessment enables clinicians to select appropriate surgical strategies and ensures that patients are adequately informed about potential intraoperative and postoperative complications.

From a surgical standpoint, the elevated risk of lingual cortical perforation observed in horizontally and deeply impacted molars, as well as in cases with parallel lingual morphology, highlights the importance of modifying the surgical approach. In such high-risk scenarios, the use of a wider mucoperiosteal flap to provide adequate visualization, careful buccal osteotomy to reduce resistance, and sectioning of the tooth to minimize the need for excessive force are advisable. Additionally, employing piezosurgery or fine burs instead of chisels or uncontrolled elevators may reduce the stress transmitted to the lingual plate. These measures can help prevent unfavorable outcomes and improve the safety profile of third molar surgery.

While this study offers valuable insights, it has certain limitations. First, its retrospective design inherently carries the risk of missing data and restricts the ability to establish causal inferences. Second, a potential selection bias should be considered, as CBCT imaging was performed only in patients for whom it was clinically indicated, which may not represent the broader population of individuals with impacted mandibular third molars. Finally, the study did not directly correlate CBCT findings with clinical outcomes such as intraoperative complications, thereby limiting the ability to translate radiological observations into definitive surgical risk predictions. Future prospective studies conducted in larger and more diverse populations, integrating radiographic, clinical, and surgical data, are warranted to validate and extend these results.

## 5. Conclusions

This study highlights the significance of anatomical variables—particularly tooth angulation and impaction depth—in influencing lingual bone morphology and the risk of cortical perforation in impacted mandibular third molars. Our findings demonstrate that deeper and more angulated impactions are more frequently associated with parallel lingual cortical morphology and a higher likelihood of perforation, especially at the apex and cementoenamel junction levels. Importantly, apical perforation was observed in approximately one-third of the cases, with horizontally and deeply impacted teeth (Class II, Level C) representing the highest-risk configurations. While morphometric parameters such as cortical thickness and apex-to-canal distance provide valuable information, they appear to be insufficient as standalone predictors of perforation risk; no definitive millimeter threshold could be established, underscoring the need to interpret thickness together with angulation and impaction depth. The study also emphasizes the limitations of panoramic radiography in assessing buccolingual relationships and supports the routine use of CBCT imaging in preoperative evaluations. By identifying high-risk anatomical patterns, these findings provide practical guidance for preoperative planning, informed consent, and surgical strategies aimed at minimizing complications and improving patient safety.

## Figures and Tables

**Table 1:** Descriptive characteristics of the impacted mandibular third molars.

		*n* (%)
Side	Right	65 (54.2%) 55 (45.8%)
	Left	
Gender	Male	34 (47.9%) 27 (52.1%)
	Female	
Pell & Gregory’s Classification/ Ramus Relationship	A	45 (37.5%) 52 (43.3%) 23 (19.2%)
	B	
	C	
Pell & Gregory’s Classification/ Impaction Depth	I	46 (38.3%) 38 (31.7%) 36 (30.0%)
	II	
	III	
Winter’s Classification/Angulation	Mesioangular	33 (27.5%) 67 (55.8%) 19 (15.8%) 1 (0.8%)
	Vertical	
	Horizontal	
	Buccolingual	
Lingual Bone Morphology	Undercut	35 (29.2%) 34 (28.3%) 36 (30.0%) 15 (12.5%)
	Parallel	
	Slanted	
	Round	
Apex Position	Contact	43 (35.8%) 41 (34.2%) 36 (30.0%)
	Non-contact	
	Perforating	
Perforation at CEJ	Yes	11 (9.2%) 109 (90.8%)
	No	
Perforation at Mid-root	Yes	30 (25.0%) 90 (75.0%)
	No	
Perforation at Apex	Yes	35 (29.2%) 85 (70.8%)
	No	

CEJ cementoenamel junction.

**Table 2:** Comparison of parameters according to Pell & Gregory’s classification (ramus relationship).

		Available Space (Concerning Ascending Mandibular Ramus)			
		Class I (*n* = 45)	Class II (*n* = 52)	Class III (*n* = 23)	
		*n* (%)	*n* (%)	*n* (%)	*p*
Lingual Bone Morphology	Undercut	19 (42.2%)	11 (21.2%)	5 (21.7%)	0.020 *
	Parallel	5 (11.1%)	18 (34.6%)	11 (47.8%)	
	Slanted	14 (31.1%)	18 (34.6%)	4 (17.4%)	
	Round	7 (15.6%)	5 (9.6%)	3 (13%)	
Apex Position	Contact	22 (48.9%)	13 (25%)	8 (34.8%)	0.055
	Non-contact	14 (31.1%)	17 (32.7%)	10 (43.5%)	
	Perforating	9 (20%)	22 (42.3%)	5 (21.7%)	
Perforation at CEJ	Yes	2 (4.4%)	4 (%7.7)	5 (%21.7)	0.058
	No	43 (95.6%)	48 (%92.3)	18 (%78.3)	
Perforation at Mid-root	Yes	8 (17.8%)	17 (32.7%)	5 (21.7%)	0.221
	No	37 (82.2%)	35 (67.3%)	18 (78.3%)	
Perforation at Apex	Yes	7 (15.6%)	22 (42.3%)	6 (26.1%)	0.014 *
	No	38 (84.4%)	30 (57.7%)	17 (73.9%)	

Chi-square test, * *p* < 0.05, CEJ cementoenamel junction.

**Table 3:** Comparison of Parameters According to Pell & Gregory’s Classification (Impaction Depth).

		Depth (Concerning the Occlusal Plane)			
		Level A (*n* = 46)	Level B (*n* = 38)	Level C (*n* = 36)	
		*n* (%)	*n* (%)	*n* (%)	*p*
Lingual Bone Morphology	Undercut	18 (39.1%)	10 (%26.3)	7 (%19.4)	0.008 *
	Parallel	4 (8.7%)	13 (%34.2)	17 (%47.2)	
	Slanted	17 (37%)	9 (%23.7)	10 (%27.8)	
	Round	7 (15.2%)	6 (%15.8)	2 (%5.6)	
Apex Position	Contact	23 (50%)	12 (%31.6)	8 (%22.2)	0.110
	Non-contact	12 (26.1%)	13 (%34.2)	16 (%44.4)	
	Perforating	11 (23.9%)	13 (%34.2)	12 (%33.3)	
Perforation at CEJ	Yes	0 (0%)	1 (2.6%)	10 (27.8%)	0.001 *
	No	46 (100%)	37 (97.4%)	26 (72.2%)	
Perforation at Mid-root	Yes	11 (23.9%)	10 (26.3%)	9 (25%)	0.968
	No	35 (76.1%)	28 (73.7%)	27 (75%)	
Perforation at Apex	Yes	11 (23.9%)	13 (34.2%)	11 (30.6%)	0.572
	No	35 (76.1%)	25 (65.8%)	25 (69.4%)	

Chi-square test, * *p* < 0.05, CEJ cementoenamel junction.

**Table 4:** Comparison of parameters according to Winter’s classification (Angulation).

		Angulation			
		Mesioangular (*n* = 33)	Vertical (*n* = 67)	Horizontal (*n* = 19)	
		*n* (%)	*n* (%)	*n* (%)	*p*
Lingual Bone Morphology	Undercut	8 (24.2%)	24 (%35.8)	3 (%15.8)	0.001 *
	Parallel	15 (45.5%)	7 (%10.4)	11 (%57.9)	
	Slanted	9 (27.3%)	24 (%35.8)	3 (%15.8)	
	Round	1 (3%)	12 (%17.9)	2 (%10.5)	
Apex Position	Contact	7 (21.2%)	31 (46.3%)	5 (26.3%)	0.127
	Non-contact	13 (39.4%)	19 (28.4%)	8 (42.1%)	
	Perforating	13 (39.4%)	17 (25.4%)	6 (31.6%)	
Perforation at CEJ	Yes	2 (6.1%)	1 (1.5%)	7 (36.8%)	0.001 *
	No	31 (93.9%)	66 (98.5%)	12 (63.2%)	
Perforation at Mid-root	Yes	13 (39.4%)	14 (20.9%)	3 (15.8%)	0.079
	No	20 (60.6%)	53 (79.1%)	16 (84.2%)	
Perforation at Apex	Yes	14 (42.4%)	17 (25.4%)	4 (21.1%)	0.145
	No	19 (57.6%)	50 (74.6%)	15 (78.9%)	

Chi-square test, * *p* < 0.05, CEJ cementoenamel junction, since there is only 1 tooth in the buccolingual position, it was excluded from the comparison.

## Data Availability

The raw data supporting the conclusions of this article will be made available by the authors on request.

## Abbreviations

The following abbreviations are used in this manuscript:

CBCT	Cone Beam Computed Tomography
DICOM	Digital Imaging and Communication in Medicine
CEJ	Cementoenamel Junction
